# Effect of Radio Frequency Energy for Intervention Processing on the Quality of Intact Eggs

**DOI:** 10.3390/foods13213457

**Published:** 2024-10-29

**Authors:** Daniela Bermudez-Aguirre, Joseph Sites, Joshua Carter, Joseph Uknalis, Brendan A. Niemira

**Affiliations:** 1 Characterization and Interventions for Foodborne Pathogens Research Unit, Eastern Regional Research Center, Agricultural Research Service, United States Department of Agriculture, 600 East Mermaid Lane, Wyndmoor, PA 19038, USA; 2 Family and Consumer Sciences, North Carolina Agricultural and Technical State University, 1601 East Market St., Greensboro, NC 27411, USA; 3 Microbial and Chemical Food Safety Research Unit, Eastern Regional Research Center, Agricultural Research Service, United States Department of Agriculture, 600 East Mermaid Lane, Wyndmoor, PA 19038, USA

**Keywords:** eggs, radio frequency, food processing, food quality

## Abstract

During conventional pasteurization, eggs are submerged for 60 min at 56.7 °C, a lengthy and costly process that affects egg quality. Radio frequency (RF), a means to pasteurize eggs without affecting quality, is a novel option based on fast volumetric heating; however, there is scarce information about the quality of such treated food. This research consisted in a comprehensive quality study on eggs treated with RF. The RF system was operated at 40.68 MHz, 40 W and 16 W (8 min total), and 42 rpm. The quality assessment included the determination of Haugh unit, yolk index, compression strength, albumen turbidity, albumen and yolk pH, and yolk color. Additional analyses were conducted to quantify the mineral composition of the eggshell (40.68 MHz, 40 W, 42 rpm, 5.5, 8.5, and 10 min); these samples were observed by SEM. The results showed that RF did not significantly (*p* > 0.05) change any quality parameters. The mineral composition remained constant in processed eggs. The SEM images of RF-treated eggs showed some smooth spots; however, these areas could exist due to the high variability of the eggshells. RF is an option to process intact eggs, maintaining their fresh quality and keeping the integrity of the eggshell to ensure the food safety of the internal egg components.

## 1. Introduction

Eggs are one of the best sources of protein in the human diet [1]; they contain high-quality protein, vitamins, minerals, essential fatty acids, phospholipids, sphingomyelin, lutein, zeaxanthin, antioxidants, and choline [2,3,4]. Eggs are widely distributed and consumed regardless of the geographic location. Egg production worldwide in 2022 was reported at 87 million metric tons; the egg consumption per person only in the United States was 281.3 units in 2023 [5]. However, one of the main food safety risks in eggs is the presence of *Salmonella* spp., which has been reported in several foodborne outbreaks worldwide, being *S. enterica* serotype Enteritidis the most common specie associated with eggs [1,6]. *Salmonella* spp. can be present inside or outside the eggshell and compromise the food safety of the product. The conventional pasteurization method for intact eggs keeps eggs submerged in hot water (56.7 °C) for 60 min, resulting in the inactivation of *Salmonella* cells inside the egg, but with undesirable changes in quality, such as a cloudy albumen [1]. Less than 3% of the U.S. production of eggs is sold pasteurized, and many countries have zero pasteurized eggs in the market [7]. The lengthy process, additional cost, and changes in quality keep egg processors away from pasteurization.

One of the main challenges during pasteurization, besides the effective inactivation of *Salmonella* cells, is to preserve the egg quality from the eggshell throughout the egg white and yolk. Most of the egg composition is protein (58% egg white), followed by fatty acids (31% egg yolk) and the eggshell (11% crust). The eggshell regulates the exchange of gases and water from inside the egg with the surrounding environment and contains internal and external shell membranes. The mineralized shell contains 96% of calcium carbonate; the rest includes Mg, P, and some trace minerals [8]. The eggshell represents a barrier between the environment and the internal contents of the egg. About 8–10% of the eggshells are damaged during commercial handling, representing economic losses and a food safety risk [9]. On the other hand, the egg white or albumen contains proteins such as ovalbumin, ovotransferrin, ovomucoid, ovomucin, and lysozyme [10]. The egg yolk also contains some proteins in a minor concentration, essential fatty acids, and bioactive compounds such as carotenoids. So, it is essential to maintain the quality of the egg during processing to preserve the nutrients of this important food.

Some novel technologies such as irradiation, microwave, and radio frequency have been explored for egg and egg product processing [1,6,11]. Microwave and radio frequency share a similar working principle, but each one works at a different frequency. Both technologies use the thermal and dielectric properties of the food to generate volumetric heating. When the product is placed in an active electric field, the water molecules inside rotate to align with the electric field. This molecular rotation generates friction that is observed as heating [12]. Because the heat is produced internally in the food and is not applied from an external source (i.e., water, air, steam) like in traditional pasteurization, the processing times are usually shorter, and the quality of microwave- and radio frequency-processed foods is better [12,13,14,15,16]. The main challenge of using microwave to pasteurize eggs is the non-uniform heating inside the eggshell because of the different water concentrations of the egg components [17]. A patent for a microwave pasteurizer for eggs was reported in South Africa, with a 2–5 log reduction in microorganisms; however, its main limitation is the penetration depth of the microwave energy [18]. Radio frequency is a novel thermal technology that has shown positive results in microbial inactivation of foodborne pathogens such as *Salmonella* spp. However, most radio frequency research has been conducted in low-moisture foods [19,20,21,22,23]. Radio frequency is still in early development for the pasteurization of high-moisture foods [14] because of the non-uniform heating in the product [24]. Previous research was reported using radio frequency and heat in eggs with the first generation of a USDA ARS-patented RF system [18,25,26,27]. However, the direct effect of radio frequency energy alone has not been fully explored in intact eggs.

This research aimed to conduct a comprehensive exploratory study on egg quality when processed with a novel technology, radio frequency. The overall quality assessment was conducted using RF energy to ensure that this technology does not affect the egg quality parameters, the mineral composition, or the structure of the eggshell.

## 2. Materials and Methods

### 2.1. Eggs

Large white eggs grade AA (4 batches, 30 dozen eggs, each) were used for this research. Eggs were from a local farm in PA and were kept refrigerated after arrival. Each egg was visually checked for cracks on the eggshell, and those with physical damage were discarded. The eggs were manually weighted and sorted out according to their weight. To study the egg quality for this project, the chosen eggs ranged from 58 to 61 ± 0.05 g (55% of the total population). The eggs were kept under refrigerated conditions (4 °C) until the day of the experiment.

### 2.2. Radio Frequency Processing

The RF system used in the present research is a new system developed from the formerly reported USDA–ARS-patented RF unit [25,26,27]. The new generation of the RF system was described in detail previously [28], and a brief description will be presented in this manuscript. The new RF system (40.68 MHz) can process up to 24 eggs at the same time. The internal temperature of the eggs was raised to 35 °C using a water bath to start the RF processing at a uniform temperature and simulate the temperature of the eggs after removing them from the hatcheries. The temperature of the albumen was controlled using water spray from individual nozzles while the eggs were rotating (42 rpm). The electrodes (5 cm × 2.5 cm × 0.2 cm) were held by nylon paddles, and gap distance between them was of about 4.32 cm. The matching network had an internal capacitor of 43 pF, and the inductor was 128 nH. This network was optimized using the Smith chart to reduce any reflected power using a vector impedance analyzer TE3000 (TrewMac Systems, Morphettville, SA, Australia). The absorbed power was recorded as 98–99%. Fiber optic probes were used to monitor the temperature inside the eggs during processing.

**Figure 1:**
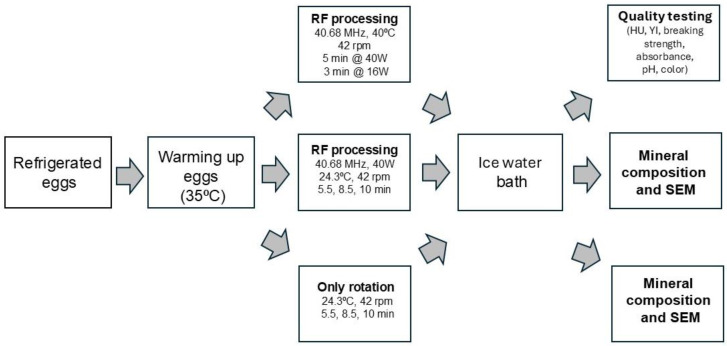
Flowchart of the three processes tested in intact eggs. RF: radio frequency, HU: Haugh unit, YI: yolk index, SEM: scanning electron microscopy.

The first set of experiments was focused on studying the egg quality after processing with RF energy. The system was operated at 40.68 MHz, and the processing consisted of 5 min at 40 W followed by 3 min at 16 W, using tap water (40 ± 0.1 °C; 14 L/min) to control the egg’s internal temperature (Figure 1). Based on preliminary trials of several experiments the second set of experiments was focused on studying the mineral composition and the effect on the eggshell structure and was conducted at 40.68 MHz, 40 W, and water spray temperature 24.3 ± 0.5 °C, and the eggs were processed with RF for 5.5, 8.5, and 10 min. The same treatments were performed inside the RF system without applying the RF energy, while only rotating the eggs, with the same processing times (5.5, 8.5, and 10 min) to see if rotation affected the eggshell structure. After processing, the eggs were cooled down using an ice water bath and dried with paper towels for further analysis (Figure 1).

All the conditions reported in this manuscript were chosen based on several trials, allowing for the identification of those processing parameters that maintain the quality of fresh eggs, with a potential to pasteurize eggs (i.e., increase temperature without quality changes) and achieve maximum absorbed power (i.e., minimal or no energy losses).

### 2.3. Egg Quality

The overall egg quality was studied using parameters such as Haugh unit, yolk index, eggshell breaking strength, albumen absorbance, yolk and albumen pH, yolk color, and mineral content. At least fifteen eggs were evaluated for each parameter.

#### 2.3.1. Haugh Unit

To measure the Haugh unit, the egg was cracked with a stainless-steel commercial egg cracker, and the egg contents were placed on top of a square glass (30 cm per side). The eggs were carefully reviewed after that to ensure the vitelline membrane was intact. The Haugh unit was measured in 3 positions (thick albumen) with a Haugh meter S-8400 (Ames Incorporated, Framingham, MA, USA).

#### 2.3.2. Yolk Index

Equation (1) was used to evaluate the yolk index (*YI*), measuring the yolk height (*a*) and the yolk diameter (*b*) with a digital caliper. The egg sample used for the Haugh unit was also used to calculate the yolk index.

*YI* = (*a*/*b*)^2^ (1)

#### 2.3.3. Eggshell Breaking Strength

The eggshell breaking strength was evaluated as previously described [28]. Briefly, a TA.XT plus texturometer (Texture Stable Micro System, Surrey, UK) was used to evaluate the shell breakage strength (*g*-force) using a compression test to simulate the cracking of the eggshell.

#### 2.3.4. Albumen Absorbance

Albumen absorbance was assessed in control and RF-processed eggs to evaluate any degradation during processing. The methodology was described in detail previously [28]. An egg cracker broke the egg, and the albumen was separated and placed in a beaker. The albumen was mixed and degassed, and absorbance was read at 600 nm in a spectrophotometer (Model 83057-06, Cole Parmer, East Hills, IL, USA).

#### 2.3.5. Yolk and Albumen pH

Fresh new and processed samples were used to measure the pH. Each egg component (yolk, albumen) was separated and homogenized by hand using a spatula. The pH was measured by direct immersion of the electrode (21 °C) with a Mettler Toledo pH meter (Columbus, OH, USA).

#### 2.3.6. Yolk Color

The yolk color is a very important quality attribute during egg processing and was evaluated using the Hunter color parameters (L*, a*, b*). The three-color parameters were measured on three different positions of the yolk using a colorimeter JZ-300 (Shenzhen Kingwell Instrument, Co, Ltd., Shenzhen, China). These parameters were used to calculate the net color difference (Δ*E*), the chroma saturation index (*C*), and the hue angle (*h*) as described previously [28].

#### 2.3.7. Mineral Content

The mineral content of the eggshell was quantified in the samples prepared for electron microscopy using energy-dispersive X-ray spectrometry. The mineral content reported for the samples was Ca, P, and Mg (%) because these represent the main minerals in eggshells.

### 2.4. Scanning Electron Microscopy

Samples of eggshells were prepared to be observed by scanning electron microscopy and to study if the effect of RF energy or rotation might affect the surface of the eggshell. Eggshell samples from control, RF-processed, and only-rotation eggs were taken, and small fragments were prepared inside a cell culture plate with 2 ml of 2% (*v*/*v*) glutaraldehyde solution overnight. Next, the solution was removed, and the samples were then covered with 2.5% glutaraldehyde (Electron Microscopy Sciences, Hatfield, PA, USA) and allowed to be fixed for 30 min. The samples were then rinsed twice for 30 min each with 2–3 mL of 0.1 M imidazole buffer (Electron Microscopy Sciences, Hatfield PA, USA), followed by 30 min intervals in 2–3 mL of 50, 80, and 90% ethanol (The Warner-Graham Company, Cockeysville, MD, USA). The samples were washed and held thrice in 2 mL of 100% ethanol before being critically point-dried. They were stacked in a wire basket, separated by cloth, and placed in a Critical-Point Drying Apparatus (Denton Vacuum, Inc., Cherry Hill, NJ, USA), using liquid carbon dioxide (Welco Co, Allentown, PA, USA) for approximately 20 min. Then, the samples were mounted on stubs and sputtered gold-coated for 1 min (EMS 150R ES, EM Sciences, Hatfield, PA, USA). They were viewed with an FEI Quanta 200 F Scanning Electron Microscope (Hillsboro, OR, USA) with an accelerating voltage of 10 kV in high-vacuum mode.

### 2.5. Statistical Analysis

Each experiment (quality assessment or electron microscopy) was conducted in different weeks and in duplicate. Microsoft Excel (version 2408) was used to conduct basic statistical analysis, and one-way ANOVA (α = 0.05) was useful to find significant differences in the quality parameters.

## 3. Results

### 3.1. Haugh Unit, Yolk Index, Compression Strength, and Absorbance

**Figure 2:**
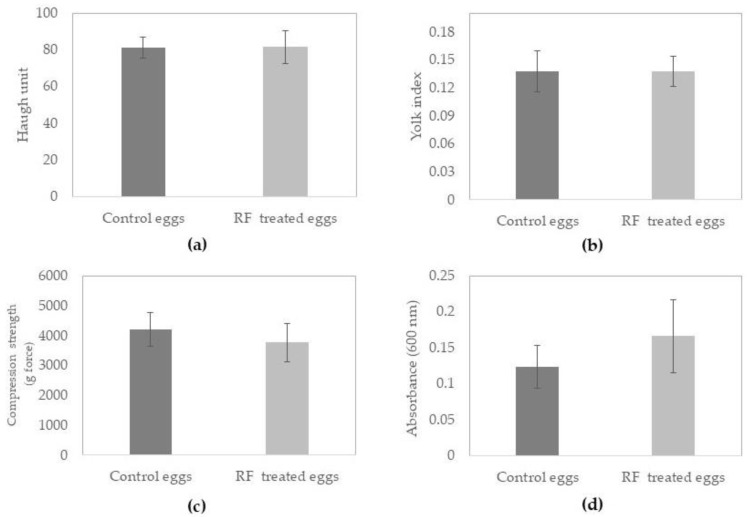
Quality attributes for control and radio frequency (RF)-processed eggs: (**a**) Haugh unit, (**b**) yolk index, (**c**) compression strength, (**d**) albumen absorbance. RF processing conditions: 40.68 MHz, 5 min at 40 W + 3 min at 16 W, water spray temperature, 40 °C, 42 rpm. *n* = at least 15 eggs were evaluated for each parameter. No significant difference (*p* > 0.05) between control and RF-processed eggs was found for any of the parameters shown.

The results of Haugh unit, yolk index, compression strength, and absorbance are presented in Figure 2a, Figure 2b, Figure 2c and Figure 2d, respectively. The Haugh unit is a common measurement in the egg industry that is related to egg freshness and quality and is based on egg weight and the height of the thick albumen [29]. Fresh eggs from young hens usually have an HU above 85; this value decreases as the egg ages [7]. A firm albumen has a Haugh unit of 72 or higher and describes eggs classified as grade AA [30]. The control eggs for this research had an average Haugh value of 81.3, and the RF-processed eggs had a final value of 81.4 (Figure 2a); there were no significant differences (*p* > 0.05) in Haugh unit after processing.

The yolk index also measures egg freshness, and its value decreases during storage because of water loss. During storage, the egg experiences several changes, not only the loss of water but also the loss of elasticity of the vitelline membrane and the liquefaction of the yolk. The water from the albumen diffuses into the yolk, and the yolk flattens [31,32]. This research showed that the yolk index remained unchanged when comparing control eggs and RF-processed eggs (0.138; Figure 2b); yolk index values below 0.28 are considered to correspond to regular eggs [33].

There are several methods to measure the eggshell breaking strength, such as the measurement of the impact fracture force or compression, and the result provides the minimum force to cause failure, such as shell cracks or breaks [34]. In this research, the compression strength of the eggshell was tested after processing with RF to ensure that there were no changes during processing that might compromise the food safety of the egg. The compression strength for the control eggs was on average 4202.29 gf, and that for the RF-treated eggs showed a slightly lower value of 3763.73 gf (Figure 2c). However, the samples had no significant differences (*p* > 0.05). The thickness of the eggshell is the main factor that contributes to the mechanical strength of the eggshell [8]. The eggshell is a barrier that prevents the egg contents from being contaminated with microorganisms and allows for the exchange of gases and moisture [35]. It has been reported that heat stress can increase the eggshell breakage and reduce the eggshell thickness [36].

The albumen quality is a very important characteristic of fresh eggs. Any changes in albumen turbidity mean that the protein has been denaturized, and then the functionality of the albumen might be affected. It has been reported that during conventional thermal pasteurization, the albumen becomes cloudy because of the effect of heat on heat-sensitive protein, leading to denaturation, aggregation, and coagulation [1,11,37]. The research presented in this manuscript shows the effect of RF heating for a few minutes; the absorbance in the samples changed from 0.123 (control eggs) to 0.166 (RF-treated eggs). Even though there was a slight increase in absorbance after RF processing, there was no significant difference (*p* > 0.05) in the values (Figure 2d).

### 3.2. pH and Color

Regarding the pH of the albumen and the yolk, the values are presented in Table 1. For both egg components, minor variations in this parameter with respect to the control samples were observed after RF processing, which were not significant (*p* > 0.05). Illera et al. [38] reported pH values for albumen and yolk of 9.45 and 6.10, respectively, in fresh eggs, which are similar to the ones found for fresh eggs in the present research (8.97 and 6.25); the differences represent natural variability in eggs. pH values are important for eggs because an increase in these values represents undesirable changes inside the egg, such as the hydrolysis of carbonic acid with the subsequent release of moisture and CO_2_ through the pores of the eggshell, a common problem observed during the storage and aging of the product [39]. Lower pH values in albumen and yolk are generally associated with egg freshness [40].

The color of the egg yolk is not only a sensory characteristic that the consumer is looking for in fresh eggs. The characteristic yellow color of the egg yolk represents a good source of carotenoids. So, the yolk color was tested after RF processing to ensure that quality was preserved through the processing. The L*, a*, and b* color parameters showed minor changes after processing, as observed in Table 1, and there were no significant differences (*p* > 0.05) compared to the control samples. b* is the color parameter that relates to the yellowness-blueness of the sample; the control egg showed a b* value of 26.68. Meanwhile, the RF-processed eggs showed a b* value of 21.93. The net change of color was 6.44; low values for this color function represent color changes that are not perceptible to the human eye [41]. The hue angle (*h*) function did not show significant differences (*p* > 0.05); this color function is commonly used as a quality indicator. The values in the present research were between 26.88° (control) and 22.50° (RF-treated eggs); the hue angle changed from 0° (reddish) to 90° (yellowish), meaning that the yolk showed an orange hue. Meanwhile, the chroma (*C*) function shows the degree of saturation, purity, or intensity of color [42] and did not show significant changes for the egg yolk after processing. It can be mentioned that the general appearance of the RF-treated eggs remained without noticeable changes after processing, and the eggs looked just like the raw, control, fresh eggs.

### 3.3. Mineral Composition

**Figure 3:**
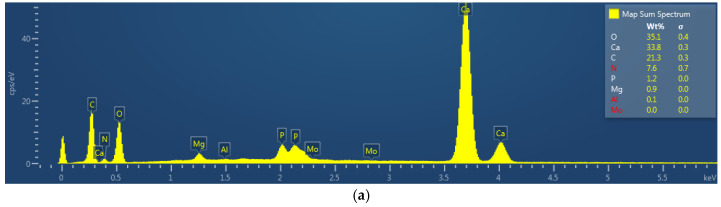

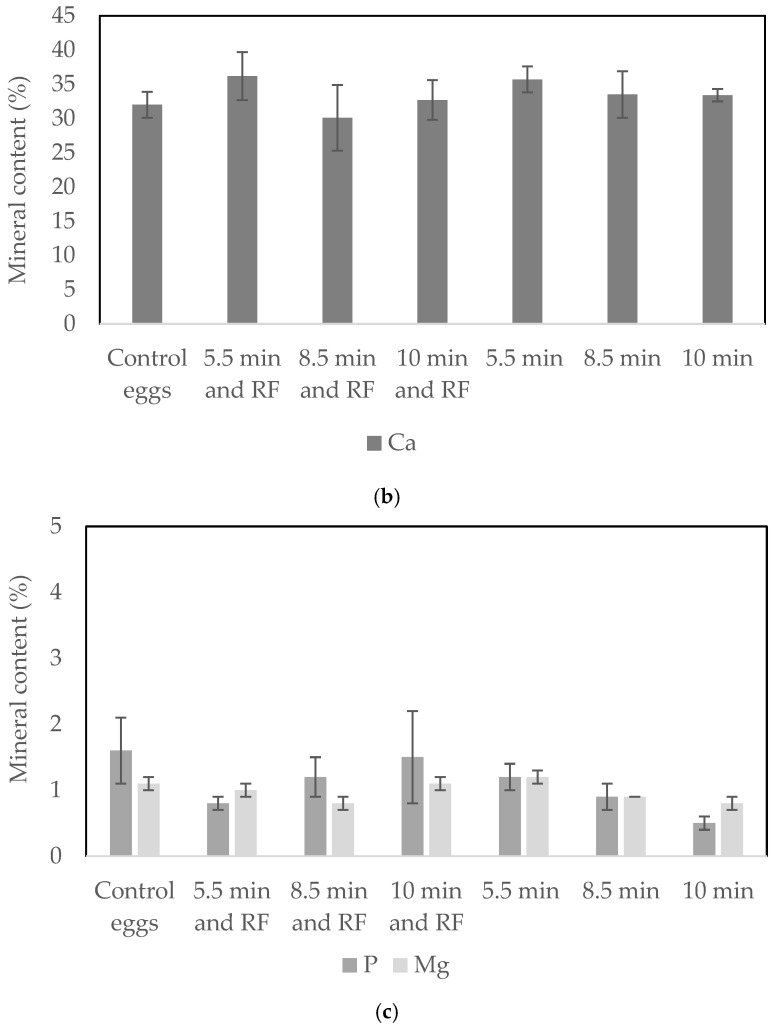
Mineral content of control eggs, radio frequency-treated eggs, and rotation-only eggs. (**a**) EDX spectra of a sample, (**b**) Ca, (**c**) P and Mg. Processing conditions: radio frequency (RF): 40.68 MHz, 40 W, water spray temperature, 24.3 °C; 42 rpm. No significant differences (*p* > 0.05) were found between control and processed samples for each mineral.

The eggshell is a natural network containing calcium carbonate, calcium phosphate, and magnesium carbonate crystals, besides some organic components and water [35]. Some innovative technologies such as pulsed electric fields (PEF), high-intensity pulsed electric fields (HIPEF), and electrical discharge-assisted mechanical milling (EDAMM) have been successfully utilized to extract calcium from eggshells and use it for other applications such as calcium supplements [35]. Although RF is based on the dielectric properties of the egg to promote volumetric heating inside the egg, the results presented in Figure 3 regarding the mineral content of the eggshell show no significant differences (*p* > 0.05) in the Ca, P, and Mg content between the control and the processed egg samples. The mineral content of the eggshells varied across the samples, being apparently unrelated to the treatment. Ca ranged from 36.3 to 30.1%, P varied from 1.6 to 0.5%, and the Mg content was from 1.2 to 0.8%. The average Ca content reported for white eggs is 34.12% [35], within the range of the minerals found in this study.

### 3.4. Scanning Electron Microscopy

**Figure 4:**
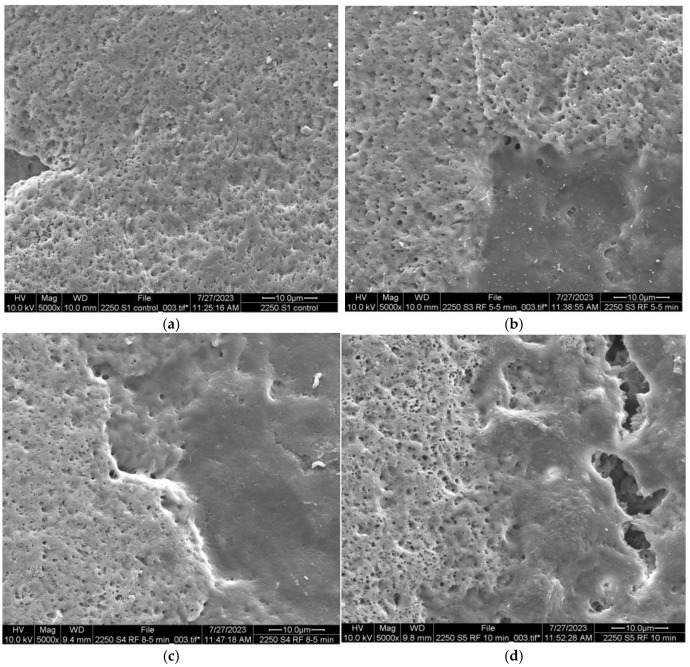

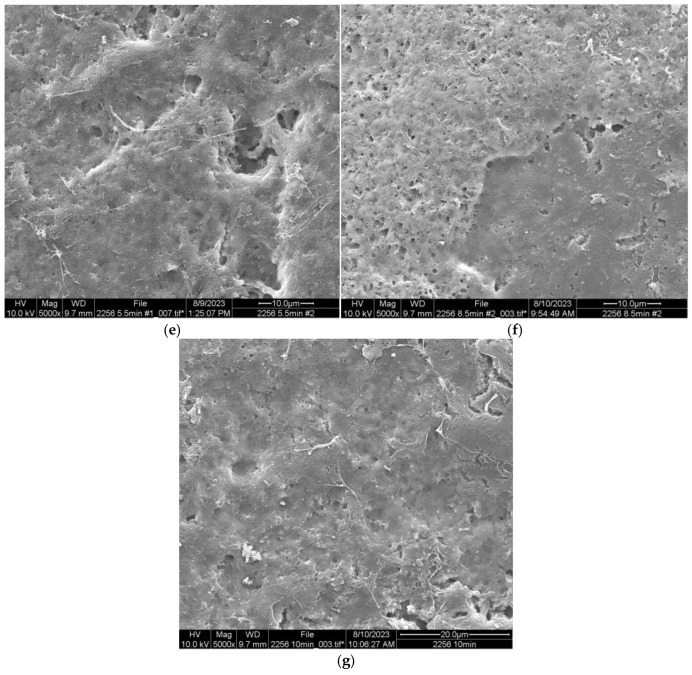
Scanning electron microscopy images of eggshells under different treatments: (**a**) Control eggs, (**b**) radio frequency-treated eggs, 5.5 min, (**c**) radio frequency-treated eggs, 8.5 min, (**d**) radio frequency-treated eggs, 10 min, (**e**) rotation only, 5.5 min, (**f**) rotation only, 8.5 min, (**g**) rotation only, 10 min. Radio frequency processing conditions: 40.68 MHz, 40 W, water spray temperature, 24.3 °C, 42 rpm.

Finally, some samples of the eggshell were observed under a scanning electron microscope to study any changes in the structure of the eggshell after RF processing. Because the system uses rollers to move the eggs, and the product is held by electrodes during processing to ensure a better temperature distribution, the rotation effect was assessed under the microscope. Several images were evaluated under the microscope; some are presented in Figure 4. The control sample showed a surface with high porosity, characteristic of eggshells (Figure 4a). The following images in Figure 4b–d show the eggshells after RF processing for 5.5, 8.5, and 10 min, respectively. Some smooth areas were observed in the eggshell samples compared to the control eggshell. Meanwhile, the last images in Figure 4e–g show the eggshells after applying the rotational movement inside the RF system but without any RF energy applied, for 5.5, 8.5, and 10 min, respectively. In these last images, the absence of porosity and the predominance of smooth patches are more observable on the egg surface. However, it is difficult to conclude that the effect of RF energy or the rotational movement affected the eggshell surface. Each egg is unique in all characteristics, and the surface widely varies from unit to unit and from the top to the bottom of the same eggshell. It was reported that eggshell porosity decreases from the egg flat area to its pointed end [36]. However, the eggshell structure is also influenced by the bird’s breeding, diet, age, and season [43]. However, considering no changes in the mineral composition and minor changes in the compression strength necessary to break the eggshell, it can be assumed that these smooth surfaces, if created by RF or rotation, did not affect the integrity of the eggshell. A previous study using cold plasma for eggshell disinfection showed some modification on the egg surface exactly in the area where the electrodes were in contact with the egg, which could be due to plasma active species or the electric field [44]. Further research is required to study the interaction between RF electrodes and eggshell in more detail. Some recommended methods to study changes in the eggshell are ultrastructural analysis, crystallographic texture analysis, and quantification of the matrix proteins; however, these are expensive and difficult to perform [9].

The results from this research open the possibility of designing an RF process to pasteurize intact eggs. Although no microbial inactivation is reported in this manuscript, the information regarding the effect of radio frequency on egg quality shows that this technology does not change any of the egg quality parameters such as Haugh unit, color, or albumen. The intelligent combination of radio frequency and a mild thermal treatment can lead to a pasteurization process [28]. This research is one of the few published works on radio frequency application on high-moisture foods, mainly intact eggs. The next step for the development of this RF system includes the scale-up of the system, the microbial validation of an RF-based pasteurization process, a final quality assessment, and further commercialization.

## 4. Conclusions

Eggs were processed with RF energy, and their overall quality was assessed and compared to that of fresh eggs. The basic quality parameters of eggs, such as Haugh unit, yolk index, compression strength, and albumen turbidity, were not changed after RF processing. Additional studies on albumen and yolk pH and yolk color showed no effect of RF on these properties. The mineral composition of the eggshell was also quantified after processing, and the content of the main elements Ca, P, and Mg remained unchanged, with no significant changes between the fresh and the RF-processed eggs. A few changes were microscopically observed for the RF-processed and rotated eggs, such as smooth areas, but these could be part of the high variability between each eggshell. This research showed that a high-moisture food, like fresh, intact eggs, can be processed with this technology without changes in quality when the temperature of each component is controlled. However, the present system was developed and patented to process intact eggs. Further research is required to test RF in other high-moisture foods, and a specific system should likely be developed. In general, RF is a technology that can process eggs without affecting the quality of the product, and the fast volumetric heating can be potentially used together with a mild thermal treatment to achieve pasteurization standards.

## Figures and Tables

**Table 1:** pH, color parameters, and functions of egg yolk after radio frequency processing ^1^.

Sample	pH Yolk	pH Albumen	L*	a*	b*	ΔE	Hue Angle (°) (*h*)	Chroma (*C*)
Control eggs	6.25 (±0.29)	8.97 (±0.10)	44.51 (±3.35)	1.17 (±3.79)	26.68 (±4.24)		26.88 (±4.36)	0.49 (±1.69)
RF-treated eggs	6.29 (±0.15)	8.94 (±0.14)	40.96 (±3.38)	−0.36 (±6.06)	21.93 (±3.37)	6.44 (±0.28)	22.50 (±3.19)	−0.48 (±1.59)

^1^ Mean values ± standard deviation. *n* = at least 15 eggs were tested for each measurement. No significant differences (*p* > 0.05) were observed between control and RF-treated eggs. RF processing conditions: 40.68 MHz, 5 min at 40 W + 3 min at 16 W, water spray temperature, 40 °C, 42 rpm.

## Data Availability

The original contributions presented in the study are included in the article, further inquiries can be directed to the corresponding author.

## Statement

The mention of trade names or commercial products in this article is solely to provide specific information. It does not imply a recommendation or endorsement by the U.S. Department of Agriculture. USDA is an equal opportunity provider and employer.
